# Comment on Uzun Ozsahin et al. COVID-19 Prediction Using Black-Box Based Pearson Correlation Approach. *Diagnostics* 2023, *13*, 1264

**DOI:** 10.3390/diagnostics14222528

**Published:** 2024-11-12

**Authors:** Manuel Graña, Goizalde Badiola-Zabala, Guillermo Cano-Escalera

**Affiliations:** Computational Intelligence Group, Computer Science Faculty, University of the Basque Country (UPV/EHU), 48013 Bilbao, Spain; ccpgrrom@gmail.com (M.G.); guillermo.cano@ehu.eus (G.C.-E.)

## 1. Introduction

The declaration of the COVID-19 pandemic by the World Health Organization (WHO) in March 2020 has triggered the publication of thousands of papers covering a plethora of aspects of the pandemic, from epidemiology models [1] to vaccine hesitancy management [2] and machine learning for diagnostics [3]. In this context, we focussed on the paper by Uzun Ozsahin et al., titled “COVID-19 Prediction Using Black-Box Based Pearson Correlation Approach”, which was published in *Diagnostics* [4], assessing some essential standards of scientific papers, which have become more critical in the medical area, and especially when treating COVID-19-pandemic-related issues. Our comments are by no means a tutorial on how to write a scientific paper, but we provide short motivations for the detected issues.

As a very general introductory comment, we notice that the title contains the expression “Black-Box Based Pearson Correlation Approach”. It seems that the authors confuse so called black box modeling approaches, such as Machine Learning (ML) [5], with a traditional correlation measure [6]. In fact, the authors report the application of linear regression and artificial neural networks for the prediction of cases and mortality in Greece and Israel.

Here, we provide a brief summary of the paper’s contents and claims in order to put the reader in context. The paper claims to compare the prediction of COVID-19 cases and deaths in Greece and Israel, providing some signal prediction results by an artificial neural network (ANN) and a multivariable linear regression (MLR) in Table 1, and several figures that we will discuss below. We note that, in their written results section, the authors are very imprecise about which results refer to cases or deaths, and whether they refer to Israel or Greece, the only guide is the captions of the figures. The conclusions provided are routine assertions somehow unrelated to the actual content of the paper, as we discuss below.

## 2. Elements of the Comments

In this section, we comment on diverse aspects that will be analysed in the paper, with a brief explanation and rationale for the analysis. The critical reading process consists of an examination of the salient features of each aspect in the paper under scrutiny, highlighting the deviations from good practices found.

### 2.1. ML Methodology

There is already a widely accepted consensus on the standard methodological elements that must be contemplated in the experimentation with and reporting of ML models [7]. Here are the most relevant for the paper under scrutiny:

1. Definition of the research question that includes the ML models that will be exploited and the meaning of the dependent variables (output) to be predicted, and the motivation for this analysis.
2. Detailed description of the independent (input) variables, including the descriptive statistics of the dataset. The feature selection and/or feature extraction processes are also specified here.
3. Detailed description of data pre-processing. Including data cleaning, normalization or scaling of the data, and handling of outliers or null values.
4. Detailed description of validation approach with specification of data set partitioning for training, validation, and test. Separation of training and test is critical to avoid double dipping [8] bias in the estimation of generalization results (aka external validation).
5. Definition of the performance measures and experimental design, including the relevant statistical tests used for the analysis of the experimental results and the extraction of conclusions from the study.
6. Exhaustive report of all computational experimental results and discussions relating them to the relevant literature. Many papers include supplementary material when the size of the comprehensive report is excessive.

### 2.2. AI Writing

Since the appearance of ChatGPT (https://openai.com/blog/chatgpt accessed on 1 October 2023), the issue of the ethically correct use of this AI tool for scientific paper writing has been considered with growing concern [9]. There is an increased acceptance of its use as an auxiliary tool—some kind of spellchecking on steroids. Some editorial companies, such as Elsevier, are including an AI writing disclosing section in the manuscript submission process. However, it is good practice to check if the submitted paper has been partially or entirely generated by AI writing, as a profilactic measure. Here, we used two tools, ZeroGPT (https://www.zerogpt.com/ accessed on 1 October 2023) and turniting (https://www.turnitin.com accessed on 1 October 2023), that provide estimations of the content that is likely to be generated by AI.

### 2.3. Result Reporting and Visualization

The results report should align with the research question, the definition of performance measures, and the experimental design. The table of contents should be as clear and self-explanatory as possible. Graphs and visualizations should be intelligible and meet standard expectations. Both tables and figures should correspond directly to the text.

### 2.4. Discussion and Conclusions

The conclusion section of a scientific article is a fundamental aspect, as it serves as the end of the study and summarizes the key results and findings. Both the discussion and findings should be related to the research question and the experimental design followed.

### 2.5. Open Science

Open Science [10] is a movement that aims to make scientific research and its results more accessible, transparent, and collaborative. The following are some key principles:

- Open access to research results: papers should be open access to all readers.
- Open access to the data: the data used for the study should be accessible without limitations. This is of special importance when the conclusions of the study may influence policy makers. Specially when high impact policies are potentially harming the people.
- Open access to code: the code should be ready for third party validation of the claimed results. If the code is not functional, the claims of the papers can not be sustained.
- Reproducibility: in the case of ML studies, reproducibility encompasses access to the code and the original data in a way that allows to reproduce the claimed results. Non-reproducible studies should not be taken into consideration for policy making.
- Open peer review
- Ethical conduct of research that includes also ethical paper writing, i.e., no misleading conclusions unsupported by evidence, or the mischievous use of AI tools.

## 3. Specific Comments

In this section, we provide specific comments, structured in subsections corresponding to the ones identified above.

### 3.1. ML Methodology

#### 3.1.1. Research Questions

The research questions as stated in the abstract and repeated in the introduction are defined as follows: “*The current study aimed to (I) compare the weekly COVID-19 cases between Israel and Greece, (II) compare the monthly COVID-19 mortality cases between Israel and Greece, (III) evaluate and report the influence of the vaccination rate on COVID-19 mortality cases in Israel, and (IV) predict the number of COVID-19 cases in Israel*”. The authors claim that this research is highly original; however, their review of the literature does not support this claim. Hence, it appears that the dependent variables are the number of cases and deaths attributed to COVID-19 in the two countries. However, we do not know whether the authors are refering to the cumulative number of cases or the daily/weekly count of cases. In Figure 7, the number of cases look like a cumulative curve; however, in Figure 8, it is not evident which kind of variables the are authors dealing with. For questions (I) and (II), there is no specific measure of comparison between the countries considered in the paper. It is not explained why questions (III) and (IV) are restricted to Israel.

Searching the paper for where these research questions have been considered, we did not find any results reporting a comparison between Israel and Greece that correspond to questions (I) and (II). Table 1 provides some results of ANN and MLR regression, but it appears to be restricted to Israel, although this is not indicated in the caption of the table, which corresponds to question (IV). Figure 6 seems to consider to question (III), while the other figures do not show any comparison between Israel and Greece.In conclusion, it appears that questions (I) and (II) have been neglected by the authors.

#### 3.1.2. Dataset

The origins of the dataset are not disclosed. There is only a general reference to the Kaggle site, but no information about the original source (like the national statistics institutes of each country). The authors mention over 200,000 COVID-19 case records and 52 independent variables. No explanation is provided about the nature of the variables. Are they time series of some kind? No information is provided. Without this information, it is not possible to assess the pertinence of the selected modeling methods and results.

#### 3.1.3. Data Pre-Processing

In this study, the authors normalize the data to a customized range of [0.05, 1] without any explanation. This artificial range may introduce some variables in the results that are not considered. The authors noted the need to curate the data, mentioning, for example, the completion of missing values, but without providing details. Data manipulation may result in significant changes in the results.

#### 3.1.4. Validation

It is important to note that the validation technique for the predictive methods is not fully clear from the steps outlined in the methods section. The authors mention both a 70–30 holdout split and 4-fold cross-validation, but it is not evident which method was applied or how it was used in the computations. The results tables lack in-depth statistical details for each test. As a best practice, it is helpful to show the standard deviations of the tests to better understand the variability of the results and to enhance the validity of the evaluation of the predictive methods. Also, there is no evidence of any statistical significance test being applied to the performance measures.

#### 3.1.5. Experimental Design

The authors describe well known ANN and MLR regression methods, but the descriptions are not complete. For instance, the training of the ANN is not discussed, while other details are described in depth. The software used for each computational process is not adequately explained, there is a mixture of Matlab, R, and Excel resources, but these remain unclear. The title and introduction talk about “black box correlation based approaches” but the correlation only appears explicitly in Table 1, where the predicted and actual time series of cases in Israel are compared.

We found no experimental design comparing alternative models. The authors compare training and testing results that are of no interest to the reader, who will only be interested in the testing results. Figures 7 to 10 are of no interest to the reader, who is usually well aware that training and testing results should differ. If the authors want to discuss overfitting, it does not appear in the paper. Figures 9 and 10 are difficult to interpret and do not correspond to any conventional experimental design.

The authors have caused some confusion regarding the meaning of the measures. In several places, the authors reported that the two ML models obtained 94% and 98% accuracy. These values correspond to the R values that appear in the table of model results, i.e., the correlation between the predicted and actual time series. Accuracy and correlation are not equivalent concepts. Looking at Figures 7 and 8, it is clear that the predicted and actual time series follow the same trend, and hence, have a high correlation; however it is clear that the prediction error is substantial. So, the authors are jumping to conclusions that are not supported by the results.

#### 3.1.6. Results Reporting

The results are reported in one table and ten figures. The table contains the results of the regression performance measures, including the correlation between the predicted and actual time series of cases in Israel. There is no corresponding table for mortality results. As explained above, no statistical significance test is provided for any value reported in the table, suggesting that model adjustment may have been based on a single run.

Figures 2 to 5 appear to serve as descriptive tools but seem irrelevant to answering any of the research questions. The figure captions do not provide any useful information about the plot contents. While they may attempt to report on variable correlations, they are flawed for the following reasons:

1. Assume that each row contains some correlation values (normalized as percentages) among variables, as identified by the color code at the bottom; there is no way to ensure that the sum of the considered correlation will be 100%. It appears that, to compensate, the authors represent the missing percentage as a form of self-correlation (a bar segment in the same color as the variable), which is a highly irregular procedure.
2. The “dependent” variables seem to be the cumulative cases and deaths in Israel and Greece, with a plot for each combination. However, the “independent” variables are weekly counts that go up and down, while the dependent variables always go up (monotonically increasing). Hence, computing the correlation between these time series has no meaning. Despite this, the authors report positive correlations in almost all pairs of variables (if our interpretation is correct).
3. For the case plots (Figures 2 and 4), the independent variables are hospital admissions and intensive care unit (ICU) admissions. These variables are, in fact, dependent of the number of cases. In other words, there is a causative link between (weekly) cases and these variables, as the cases may become (with some delay) hospital admissions and the hospital admissions may become ICU admissions. Hence, using them as explicative variables for the (total) cases is senseless.
4. For the death plots (Figures 3 and 5), the independent variables are the death count variables, which is perfectly senseless. The ”total deaths” and “total deaths per million” are perfectly correlated variables; in fact, they are the same variable except for a scale factor. The same is true for “new deaths” and “new deaths per million”, and “new deaths smoothed” and “new deaths smoothed per million”. The correlation between these pairs should be 100%, but the authors report other values. In any case, only one pair should be used in any regression or correlation analysis. Furthermore, computing the correlation between the various death variables that have the same information is perfectly senseless.
5. Similarly, in the case plots (Figures 2 and 4) “weekly hospital admissions” and “weekly hospital admissions per million” are perfectly colinear, as are “weekly icu admissions” and “weekly ICU admissions per million”. The values in the plots appear to be completely disconnected from reality.

Figure 6 appears to attempt to address question (III) by interpreting the plot as correlation coefficients. The authors include an explanatory phrase at the end of Section 3.1 that offers a remarkable conclusion “*As demonstrated in Figure 6, there was a strong correlation between total vaccinations and total death cases in Israel. The rate of vaccination, therefore, did not influence the mortality rate*”.

Figures 7 and 8 visualize the comparison between the predicted and actual MLR time series of cases for the training and testing data, respectively, without specifying the country. It can be appreciated that the predicted and actual time series do not match, so the prediction error should be significantly high but there is no specific statistical test. Figure 7 confirms that the actual case time series is cumulative. The units of the x-axis and y-axis are not specified (day?, cases?). In Figure 8, the cumulative nature of the case variable is not very evident, possibly due to rendering effects that attempt to create a 3D appearance. The units of the x-axis and y-axis are not specified (day?, cases?), and a label “total_cases” in the x-axis adds to the confusion.

The comparison of the ANN training and testing $R^{2}$ values with the MLR $R^{2}$ value (at testing?) provided by Figure 9 has no specific scientific value. Furthermore, the mention in the caption of “clustered” is highly confusing. The authors have not carried out any clustering process. Similarly, the comparison in Figure 10 is of little interest and it is not convincingly explained in the text. The x-axis is undefined in this figure, and the y-axis labels are unclear. What is the meaning of the numbers 3 and 4 in this axis? The explanation in the text of these figures is meaningless.

### 3.2. AI Writing

The writing of abstract and introduction sections collects many common trivia about the pandemic with little relevance to the actual research question. The application of ZeroGPT over the text of the various sections carried out online (screenshots can be found in [11]) provided the following results:

1. Abstract—85.9%
2. Introduction—54.29%
3. Materials and Methods—Data 45.13%—Models 54.82%
4. Application of Results and Discussion—17.03%
5. Conclusions—0%

The application of Turnitin tools for AI writing detection estimated that 23% of the entire paper was be generated by AI. Considering these results and the relative lack of coherence in the writing, it seems that much of the writing was done by AI. As far as we know, the journal does not ask for AI writing disclosure at the time of this writing. Other than that, the paper has many awkward expressions such as “*Analyzing data helps determine the navigational and scientific value of the data*” in the first paragraph of Section 3.

### 3.3. Discussion and Conclusions

A discussion of the results is missing. The conclusions section is a collection of uninformative trivialities. However, as pointed out above, there is a strong conclusion formulated regarding Figure 6, which has been neglected in the conclusions section. We remind the reader of the literal phrase at the end of Section 3.1 of their paper “*As demonstrated in Figure 6, there was a strong correlation between total vaccinations and total death cases in Israel. The rate of vaccination, therefore, did not influence the mortality rate*”. It appears that, for some reason, the authors have overlooked this strong conclusion both in the abstract and in the conclusions section.

### 3.4. Open Science

Regarding the application of open science practices, the authors have failed in several aspects. First, the authors do not accurately specify the data source, providing only a generic URL for the Kaggle site, and they fail to link to a repository where the exact version of the data can be accessed. Furthermore, they do not provide the code used for their computations. Their description of the implementations is confusing, mentioning Matlab, R, and Excel without clarifying which tool was used for each result. It appears that figures have been created with Excel. Therefore, the works of the authors are irreproducible. Also, the authors have apparently used AI writing tools without disclosure.

## 4. Discussion and Conclusions

The critical reading contained in this comments paper should highlight that very low quality papers can be accepted and published as peer review papers. We think that this is a very critical issue for science today. However, editors and the mainstream trends in scientific/technical publications appear to favor ”being nice and politically correct” over scientific and academic correctness. The role of reviewers and academic editors is paramount in ensuring, at the very least, formal quality standards for published papers. These papers should carry out a proper scientific discussion over issues of great impact, such as it is everything related to COVID-19 pandemic, which has given a strong shock to all strata of society. A simple search in PubMed with the single term “COVID-19” provides almost 500,000 references in the very short time span since the declaration of the pandemic by the WHO. It is evident that a large army of reviewers and academic editors free from conflicts of interest should be levied to face this tsunami of papers, but quality reviewers and academic editors should also consider their own publication track, which would wither if they put too much effort and time into reviewing tasks [12]. Without touching the discussion of the demographics of the research community, it is very likely that science and scientific publication is at a critical point, which has been acknowledged for some time [13]. The paper that has been the subject of this critical reading is just another in a huge heap of scientific misinformation [14] arising from careless or compromised publication. If we must highlight something from this critical reading exercise, it is the absence of any reference to the conclusions drawn from Figure 6 in either the abstract or the conclusions section of the paper. This is no trivial matter, as many papers in the literature about COVID-19 seem to have followed this pattern. Important and ground breaking but controversial results found by the authors are relegated to the supplementary material or left out of the discussion. Oftentimes, this issue arises not from the authors’ desire or lack of knowledge. In conclusion, we call for greater systematic rigor in the review process and the freedom to clearly state the findings of the work, even if this challenges interests that are contrary to the principles of science.

## Data Availability

All materials used in this critical reading are available from [11].

## Abbreviations

The following abbreviations are used in this manuscript:

ML	Machine Leaning
AI	Artificial Intelligence
ANN	Artificial Neural Network
MLR	Multivariate Linear Regression
ICU	Intensive care unit
